# Effect of maternal BMI on labor outcomes in primigravida pregnant women

**DOI:** 10.1186/s12884-021-04236-z

**Published:** 2021-11-08

**Authors:** Eissa Khalifa, Alaa El-Sateh, Mohamed Zeeneldin, Ahmed M. Abdelghany, Mahmoud Hosni, Ameer Abdallah, Sameh Salama, Mazen Abdel-Rasheed, Hashem Mohammad

**Affiliations:** 1 Obstetrics and Gynecology Department, Faculty of Medicine, Minia University, Minya, Egypt; 2 Reproductive Health Research Department, National Research Centre, 33 El-Buhouth St, Dokki, Cairo, 12622 Egypt

**Keywords:** BMI, Obesity, Vaginal birth, Caesarean section

## Abstract

**Background:**

This study aims to detect the effects of increased BMI on labor outcomes in primigravida pregnant women.

**Methods:**

A cross-sectional study involved 600 full-term singleton primigravida pregnant women who presented in the active phase of labor to the labor ward. They were divided according to BMI into three equals groups; women with normal BMI (group I), overweight women (group II), and women with class I obesity (group III).

**Results:**

We found that high BMI was associated with a significantly increased risk of Caesarean section (C.S.) (13% in group I, 18% in group II and 40% in group III). Women with higher BMI and delivered vaginally had a significantly prolonged first and second stage of labor, consequently increased the need for oxytocin augmentation as well as the oxytocin dose. Regarding the maternal and fetal outcomes, there are significantly increased risks of postpartum sepsis, perineal tears, wound infection, as well as significantly increased birth weight and longer neonatal stay in the neonatal unit (NNU).

**Conclusion:**

Obese primigravida pregnant women were at higher risk of suboptimal outcomes. Besides, prolonged first and second stages of labor and the incidence of C.S. have also been increased.

## Background

Obesity is a major concern worldwide, with its incidence has been doubled in the last three decades. This increase represents a challenging health care problem. Obesity is now considered the most common medical problem associated with pregnancy. According to WHO estimates, in 2008, about 23% of European women are obese, and 50% are overweight [1].

Recently, there has been a rising concept that the adipose tissue is an endocrine organ as it stores and releases preformed steroid hormones, converts precursors to biologically active hormones, and converts active hormones to inactive metabolites. In addition to this, adipocytes express several enzymes critical to steroid hormone biosynthesis and metabolism [2].

Obesity in pregnancy includes pre-pregnancy and women who become obese from excess weight gain during pregnancy. Pre-pregnancy BMI can detect Pre-pregnancy obesity. Excess weight gain during pregnancy is diagnosed when the weight gained during pregnancy exceeds the recommended weight gain by the institute of medicine (IOM). The weight gain in terms of these recommendations should be between 12.5 and 18 kg if the BMI is less than 19.8 kg/m^2^. In case the BMI is between 19.8 and 26.0 kg/m^2^, the weight gain should be between 7.0 and 11.5 kg, and if a BMI > 29.0 kg/m^2^, weight gain should not exceed 7.0 kg [3].

The maternal nutritional status before and during pregnancy has a significant influence on fetal development, the health of newborn babies, and their development. Complications related to maternal obesity could be maternal such as gestational diabetes and preeclampsia, fetal such as prematurity, intrauterine fetal death, or neonatal and childhood complications [4].

The primary aim of this study was to determine the maternal and fetal outcomes in relation to maternal BMI, while the secondary outcomes were the mode of delivery, the duration of labor, duration of the first and second stages, need for oxytocin, and its dose.

## Methods

This cross-sectional study was conducted between April 2017 and March 2018. The study was approved by the Ethical Committee of the Faculty of Medicine, Minia University. This multicentric study has been carried out on women who attended the labor ward, Minia University teaching hospital, and two private hospitals in Cairo. All women participated voluntarily; privacy and confidentiality of data were assured all through the research work. After a brief explanation by an obstetrician, informed verbal consent was taken from every participant during the pain-free interval between contractions.

Women were included in this study if they fulfilled the following criteria; age between 20 and 35 years old, primigravida, single full-term pregnancy between 37 and 40 weeks, cephalic presentation, and were presented in the active phase of labor (cervical dilatation ≥4 cm with regular uterine contractions i.e.3-5 contractions/10 min). Women with any of the following criteria were excluded from this study; high-risk pregnancy, relevant medical or surgical history (e.g., D.M., scared uterus), suspected cephalopelvic disproportion (i.e., EFW > 4.5 kg, short maternal stature < 150 cm), and fetal comorbidities.

In this study, the admission BMI was used to categorize the participants. The weight and height were routinely measured for each woman coming in labor, then BMI was calculated according to the formula “weight in kg / (height in m)^2^”. Women were distributed into three groups based on their BMI according to the WHO classification of obesity; each group included 200 participants. Group I, women with normal BMI (18.5-24.9 Kg/m2), group II consisted of women who are overweight (BMI 25-29.9 Kg/m2), and the last group included women with class I obesity (BMI 30-34.9 Kg/m2). Obstetricians monitored and managed labor according to the progress documented on the partogram. Women with slow progress or irregular uterine contractions have been given oxytocin drip to augment labor if no contraindication. Cardiotocographic (CTG) fetal monitoring was continued through the labor in cases augmented with oxytocin. The decision to deliver by cesarean section was taken by the consultant obstetrician or an experienced registrar only after a full discussion with the on-call consultant.

The sample size was calculated based on a previous study comparing Caesarean section (C.S.) rate in obese and non-obese women coming into labor, which was 23.5 and 8.8%, respectively, then the power of the study was set as 90% with an alpha error of 0.05. The ratio of the study groups was adjusted to 1:1:1. Consequently, the proper sample size is 200 participants for each group. Therefore, the first 200 women who fulfill the inclusion criteria for each group were enrolled in the study.

At the end of the study, all data were analyzed using the IBM SPSS version 20 (IBM Corp, Armonk, NY, USA). Data were summarized using mean and standard deviation for quantitative variables and frequencies (number of cases) and relative frequencies (percentages) for categorical variables. Comparisons between groups were done using analysis of variance (ANOVA) with multiple comparisons Bonferroni post hoc test in normally distributed quantitative variables. On the other hand, the non-parametrical Kruskal-Wallis test and Mann-Whitney test were used for non-normally distributed variables. For comparing categorical data, a Chi-square test was performed. *P*-values less than 0.05 were considered statistically significant.

## Results

The study was carried out on 600 women who fulfilled the inclusion criteria. There was no significant difference between the three groups concerning age. Regarding the mode of birth, there was a significant difference between the three groups. Class I obesity group showed markedly increased C.S. rate due to failure to progress compared to the overweight and the normal BMI groups (40, 18, and 13%, respectively). On the contrary, there was no significant difference between the three groups regarding postpartum hemorrhage (Table 1).

**Table 1:** Comparison between groups regarding the demographic data, postpartum complications and neonatal outcomes

	Normal BMI (*n* = 200)	Overweight (*n* = 200)	Class I obesity (*n* = 200)	*P*-value
Age	28.07 ± 4.06	28.93 ± 4.37	28.50 ± 4.15	0.120
Mode of birth				
VB	174 (87%)	164 (82%)	120 (60%)	< 0.001*
CS	26 (13%)	36 (18%)	80 (40%)	
Postpartum Hemorrhage	28 (14%)	32 (16%)	43 (21.5%)	0.120
Neonatal birth weight (grams)	3245 ± 776	3637 ± 730	3789 ± 664	< 0.001*
Apgar score				
1 min	8.0 ± 0.9	7.7 ± 0.8	7.7 ± 0.6	< 0.001*
5 min	8.7 ± 0.9	8.3 ± 0.8	8.2 ± 0.7	< 0.001*
NNU admission	11 (5.5%)	14 (7%)	20 (10%)	0.220
Duration of NNU admission (days)	4.5 ± 1.9	7.9 ± 1.3	10.1 ± 2.2	< 0.001*
Intrapartum mortality	0	1 (0.5%)	1 (0.5%)	0.606
Neonatal sepsis	0	1 (0.5%)	1 (0.5%)	0.606
Neonatal mortality	1 (0.5%)	2 (1%)	3 (1.5%)	0.603

Data presented as mean ± SD or number and (%)

*CS* Caesarean Section, *NNU* Neonatal unit, *PG* Primigravida, *VB* Vaginal birth

*P-value < 0.05 is significant

From the neonatal aspect, there was a highly significant difference between all groups regarding birth weight (*P* <  0.001). Despite neonatal Apgar score in overweight and obesity groups was lower than that in the normal BMI group, there was no significant difference in the incidence of intrapartum mortality, neonatal sepsis, or neonatal mortality. Similarly, there was no significant difference between the three groups regarding the admission rate to the neonatal unit (NNU), but once admitted to the NNU, the duration of stay showed a significant difference (Table 1).

When analyzing the partograms of women who delivered vaginally (174 of normal BMI, 164 overweight women, and 120 of class I obesity), we found that there was a significant difference regarding the duration of the first and second stages, the need for oxytocin as well as its dose, the incidence of perineal tears (including cases of instrumental delivery) and the incidence of postpartum sepsis. It was clear that these variables increased in the class I obesity group compared to the other groups (Table 2).

**Table 2:** Comparison between groups regarding the data of vaginal birth

	Normal BMI (*n* = 174)	Overweight (*n* = 164)	Class I obesity (*n* = 120)	*P*-value
Duration of 1st stage (hours)	11.0 ± 1.4	11.6 ± 2.1	13.0 ± 2.1	< 0.001*
Delayed dilation	19 (10.92%)	21 (12.8%)	35 (29.17%)	< 0.001*
Need of oxytocin	59 (33.91%)	79 (48.17%)	75 (62.5%)	< 0.001*
Oxytocin dose (mU/min)	9.9 ± 2.3	10.7 ± 3.8	13.1 ± 3.6	< 0.001*
Duration of 2nd stage (min)	59.0 ± 24.8	63.5 ± 18.0	64.8 ± 19.4	0.044*
Perineal tears (2rd or 3rd degree)	2 (1.15%)	7 (4.27%)	10 (8.33%)	0.010*
Postpartum sepsis	2 (1.15%)	6 (3.66%)	18 (15%)	< 0.001*

Data presented as mean ± SD or number and (%)

*P-value < 0.05 is significant

For the 142 women who delivered via C.S. for various obstetric indications, the intraoperative duration of C.S. in the obesity group was significantly higher than that in overweight and normal BMI groups 65.6 ± 14, 54 ± 12.5 and 50.9 ± 17.9 mins respectively). Besides, wound healing, seroma formation, and wound infection showed a significant difference between the three groups. On the other hand, the difference between groups regarding the intraoperative complication, anesthetic complications, and postpartum sepsis was insignificant (Table 3). Furthermore, odd ratio analysis of the three groups regarding the mode of birth showed that the risk of birth by CS in the class I obesity group was 4.46 times higher than that in the normal BMI group and 3.04 times higher than that in the overweight group (Table 4).

**Table 3:** Comparison between groups regarding the data of Caesarean section

	Normal BMI (*n* = 26)	Overweight (*n* = 36)	Class I obesity (*n* = 80)	*P*-value
Duration of CS (minutes)	50.9 ± 17.9	54.0 ± 12.5	65.6 ± 14.0	< 0.001*
Extended angels	2 (7.69%)	4 (11.11%)	8 (10%)	0.904
Bladder injury	0	0	1 (1.25%)	0.677
Wound healing and complication				
Clean and healed	22 (84.62%)	22 (61.11%)	42 (52.5%)	0.014*
Seroma formation	2 (7.69%)	8 (22.22%)	25 (31.25%)	0.049*
Infected wound	1 (3.85%)	5 (13.89%)	20 (25%)	0.039*
Postpartum sepsis	1 (3.85%)	3 (8.33%)	17 (21.25%)	0.043*

Data presented as mean ± SD or number and (%)

*CS* Caesarean Section

*P-value < 0.05 is significant

**Table 4:** Odds Ratio for the mode of birth (Vaginal birth versus Caesarean Section)

	Group I vs. Group II	Group I vs. Group III	Group II vs. Group III
Odds ratio	1.47	4.46	3.04
95% CI	0.850 - 2.540	2.706 - 7.355	1.920 - 4.803
*P*-value	0.167	< 0.001*	< 0.001*

*P-value < 0.05 is significant

## Discussion

Obesity is a worldwide major health problem. In Egypt, 35% of the adult population suffers from obesity, representing the highest percentage in the world among this age group [5]. Obesity with pregnancy is very common and increases the obstetrical risks, as well as the possibility for CS. Egypt is ranked as the third country worldwide regarding the rate of CS (52%), which may be attributed to the high prevalence of obesity and overweight among pregnant women [6].

As obesity is a major concern worldwide, it is important to stand on its impacts on labor. In our study, it was clearly shown that obesity rather than overweight affects labor as well as the mode of birth. This was represented by a highly significant CS rate in the class I obesity group versus overweight and normal BMI groups (40, 18, and 13%, respectively). This was confirmed by the multiple regression analysis of data regarding the mode of birth, as the risk for CS birth in the obesity group was about 4.46 times that in the normal BMI group and nearly about three times that in the overweight group. Besides, the risk for CS birth in the overweight group was 1.47 times that in the normal BMI group. These findings agree with the results of previous studies that revealed increased CS rates in obese pregnant women [7, 8].

Different explanations could support our findings in this study. Carlson et al. (2017) showed that obese women received higher oxytocin doses during labor induction due to their large body volume [9]. Besides, a meta-analysis in 2018 found that maternal obesity prolongs labor progress with an increased incidence of CS due to fetal macrosomia and the increased risk of shoulder dystocia [10]. Moreover, it was found that there is an increased risk of fetal distress with maternal obesity with subsequent increased CS rates [6].

In 2017, Maged et al. studied the effect of increased BMI on labor progress in pregnant women. They showed that there was a significantly prolonged first stage as well as a significantly increased emergency CS rate in obese women. These results also agree with our results [11]. Moreover, these findings have been confirmed in a large previous analytic study, which involved 118,978 women in labor. There was a significant negative correlation between BMI and labor progress as normal BMI women gave birth 2 and 4 h earlier than obese and morbidly obese women, respectively. They suggested that the protocol of labor management should consider personal variation regarding maternal BMI [8].

Catalano and Shankar showed that women with obesity were more liable to postpartum hemorrhage, which agrees with our findings [12]. In addition, our results agree with Catalano and Shankar regarding the significant positive correlation between maternal weight and increased fetal weight [12]. Our study also showed that newborn babies of obese women tend to stay a significantly longer duration at the NNU. This was also previously presented by Meghan and Colleagues as they demonstrated that newborn babies for obese women are more liable to be macrosomic as well as suffering from other complications [13].

Our study showed that the incidence of wound healing was significantly higher in the normal BMI group than that in the overweight and obesity groups (84.6, 61.1%, and 52.5 respectively). This was also demonstrated by Simon and colleagues in their study, which included 2231 women. They found that maternal BMI of more than or equal to 30 is associated with a significantly increased risk of surgical site infection with OR equal to 4.1 (*P* <  0.001) [14].

The importance of our study arises from clearly showing that the outcomes of labor are significantly affected by increased BMI. The points of strength are being multicentric study with large sample size and being limited to a specific population, which is single full-term primigravida pregnant women, without any medical or surgical factor that may affect labor route.

The main limitation in this study is the absence of class II and III obesity groups, which may be explained by the rarity of morbid obesity among this age group as well as in primigravida women. Another limitation point is that some patients refused the use of instrumental during the trial for normal vaginal delivery; however, it did not affect our results. Also, BMI was calculated on admission to the labor ward, and not in early pregnancy, as many of those women were seen for the first time only during labor with no previous documentation of their BMI in early pregnancy.

## Conclusion

According to the results of this study, we found that BMI has a robust impact on both maternal and fetal outcomes. Obese primigravida pregnant women were at higher risk of suboptimal outcomes, including postpartum sepsis, perineal tears (in women who deliver vaginally), wound infection, increased birth weight, and longer neonatal stay in the NNU. In addition, prolonged first and second stages of labor, as well as the incidence of CS, have also been increased. Hence, counseling for weight reduction in pre-conception clinics is of crucial importance and will improve the outcomes of labor.

## Data Availability

The datasets used and/or analyzed during the current study are available from the corresponding author on reasonable request.
